# Implementing a Machine Learning Screening Tool for Malnutrition: Insights From Qualitative Research Applicable to Other Machine Learning–Based Clinical Decision Support Systems

**DOI:** 10.2196/42262

**Published:** 2023-07-13

**Authors:** Melanie Besculides, Madhu Mazumdar, Sydney Phlegar, Robert Freeman, Sara Wilson, Himanshu Joshi, Arash Kia, Ksenia Gorbenko

**Affiliations:** 1 Institute for Healthcare Delivery Science Icahn School of Medicine at Mount Sinai New York, NY United States; 2 Tisch Cancer Institute Icahn School of Medicine at Mount Sinai New York, NY United States; 3 Department of Psychology New York University New York, NY United States; 4 Hospital Administration Icahn School of Medicine at Mount Sinai New York, NY United States; 5 Clinical Nutrition Icahn School of Medicine at Mount Sinai New York, NY United States

**Keywords:** machine learning, AI, CDSS, evaluation, nutrition, screening, clinical, usability, effectiveness, treatment, malnutrition, decision-making, tool, data, acceptability

## Abstract

**Background:**

Machine learning (ML)–based clinical decision support systems (CDSS) are popular in clinical practice settings but are often criticized for being limited in usability, interpretability, and effectiveness. Evaluating the implementation of ML-based CDSS is critical to ensure CDSS is acceptable and useful to clinicians and helps them deliver high-quality health care. Malnutrition is a common and underdiagnosed condition among hospital patients, which can have serious adverse impacts. Early identification and treatment of malnutrition are important.

**Objective:**

This study aims to evaluate the implementation of an ML tool, Malnutrition Universal Screening Tool (MUST)–Plus, that predicts hospital patients at high risk for malnutrition and identify best implementation practices applicable to this and other ML-based CDSS.

**Methods:**

We conducted a qualitative postimplementation evaluation using in-depth interviews with registered dietitians (RDs) who use MUST-Plus output in their everyday work. After coding the data, we mapped emergent themes onto select domains of the nonadoption, abandonment, scale-up, spread, and sustainability (NASSS) framework.

**Results:**

We interviewed 17 of the 24 RDs approached (71%), representing 37% of those who use MUST-Plus output. Several themes emerged: (1) enhancements to the tool were made to improve accuracy and usability; (2) MUST-Plus helped identify patients that would not otherwise be seen; perceived usefulness was highest in the original site; (3) perceived accuracy varied by respondent and site; (4) RDs valued autonomy in prioritizing patients; (5) depth of tool understanding varied by hospital and level; (6) MUST-Plus was integrated into workflows and electronic health records; and (7) RDs expressed a desire to eventually have 1 automated screener.

**Conclusions:**

Our findings suggest that continuous involvement of stakeholders at new sites given staff turnover is vital to ensure buy-in. Qualitative research can help identify the potential bias of ML tools and should be widely used to ensure health equity. Ongoing collaboration among CDSS developers, data scientists, and clinical providers may help refine CDSS for optimal use and improve the acceptability of CDSS in the clinical context.

## Introduction

Machine learning (ML)–based clinical decision support systems (CDSS) are becoming ever more popular in clinical practice settings due to their ability to sift through massive amounts of data rapidly to identify those at greatest risk for an outcome of interest early when timely intervention may be possible. Despite the potential to improve patient care, ML-based systems are often criticized for being limited in usability, interpretability, and effectiveness [1,2]. Successful integration into clinical practice requires widespread, formal evaluations. Most evaluation literature to date focuses on whether ML algorithms can predict an outcome or predict it better than previous algorithms. While developing and validating algorithms is a necessary first step, evaluating the implementation of ML-based CDSS is critical to ensure that CDSS is acceptable and useful to clinicians and helps them deliver high-quality health care. Buy-in for ML-based CDSS is not universal, and often clinician input in the development, implementation, and evaluation of systems is lacking [3,4]. Clinician engagement is most prevalent in the early stages of ML-based CDSS design and late stages of implementation [4]. Understanding implementation successes and challenges can help fine-tune implementation methods prior to scale-up as well as identify unsuccessful systems to deimplement to preserve resources.

The Mount Sinai Health System, a large urban health system in New York City (New York, United States), uses several ML-based CDSS to streamline processes and increase the efficiency of care delivery. These include systems related to predicting clinical deterioration, in-hospital mortality, intensive care unit transfer, risk of falls, and malnutrition [5-9]. This study focuses on an evaluation of the malnutrition ML-based CDSS. Malnutrition is a common and underdiagnosed condition among hospital patients that can have serious adverse impacts. Untreated malnutrition can delay recovery, impair immune and organ function, and increase hospital length of stay, readmission, morbidity and mortality burden, and health care costs [10-17]. For this reason, the Joint Commission requires that all hospitalized patients be assessed for malnutrition risk [18]. The Centers for Medicare and Medicaid reimburse chronic care management for patients with 2 or more chronic conditions “expected to last at least 12 months or until the patient dies, or that place the patient at significant risk of death, acute exacerbation/decompensation, or functional decline” [19]. Therefore, diagnosing malnutrition may also have an impact on hospital reimbursement.

The Malnutrition Universal Screening Tool (MUST) is a widely used 5-step process examining BMI, unintentional weight loss, and acute disease effect [20,21]. Patients at low risk for malnutrition are given a score of 0 and receive routine clinical care, those at medium risk receive a score of 1 and are observed but not treated, and those at high risk receive a score of 2 or more and are referred for malnutrition treatment. Although MUST is a well-accepted screening tool, this and other tools used to screen for malnutrition have been suboptimal, leading to late or missed opportunities for intervention.

To improve early malnutrition diagnosis, Mount Sinai developed MUST-Plus, an ML-based application that uses electronic health record data including Epic (electronic medical record) [22], Cerner (admit discharge transfer) [23], SCC (Lab) [24], and Muse (electrocardiogram) [25] to predict patients at risk for malnutrition. MUST-Plus was first tested and refined in our largest hospital, The Mount Sinai Hospital (MSH), and proved to be superior (higher sensitivity and specificity) to the traditionally used MUST screening tool [9]. The tool’s higher accuracy led to its implementation across the 5 other hospitals in the Mount Sinai Health System over a 2-year period (2018-2020). The MUST-Plus application is integrated into the Epic [22] platform (electronic medical record used by Mount Sinai) and was improved over time at all hospitals and some hospitals have implemented 2 versions of the tool. The MUST-Plus application is part of an ML-based CDSS.

MUST-Plus risk scores range from 0 (lowest risk) to 1 (highest risk), and each hospital has set its own threshold for prioritizing patients for early assessment by a registered dietitian (RD). The threshold is based on the best performance per hospital, defined as an optimal combination of high sensitivity and an acceptable false-positive rate given staffing. The score’s interface has a hover tool that displays the top factors contributing to the predictive score, which often include BMI, albumin level, age, height, length of stay, hemoglobin, platelet count, red blood cell count, and potassium level.

## Methods

### Overview

To examine the implementation of MUST-Plus and identify best practices before further dissemination, we used semistructured qualitative interviews. The open-ended nature of this qualitative approach is well-suited to identify challenges and solutions and explore clinicians’ perspectives and areas for improvement. Specifically, we sought to identify user experiences with the tool’s output including facilitators and barriers to implementation from the perspective of RDs who use the CDSS daily.

### Data Collection

We conducted a qualitative postimplementation evaluation study with RDs who used the MUST-Plus CDSS in their everyday work. The study team approached 24 RDs across the 6 hospitals using MUST-Plus. All RDs on staff were female. RDs were purposefully selected to capture variation across staff in different hospitals, staff who have used different versions of the tool, and staff at different levels (nutritionist or clinical nutritionist or dietitian, senior dietitian, clinical nutrition coordinators, and advanced coordinator). We did not collect demographic information such as race and ethnicity or age. To gather information from other stakeholders, RDs were asked to identify other clinicians who could provide additional insight into the implementation of MUST-Plus. Because no other clinicians reportedly interacted with MUST-Plus, we focused this study on RDs.

Interviews were scheduled via Zoom (Zoom Video Communications) at times convenient for RDs and were recorded with permission. Interviews were conducted over a 2-month period by 3 coauthors (MB, KG, and SP) using a semistructured guide (see Multimedia Appendix 1) and lasted approximately 40 minutes. The researchers conducted the first interview jointly to ensure the questions were understood and gathered the desired information. The study team met weekly to discuss progress. We reached saturation [26] at interview 11, meaning each new interview was yielding very few new concepts at that point. We continued to interview 6 more RDs to ensure different hospitals were represented in our sample and to confirm saturation. Interview recordings were transcribed using Temi web-based software, verified for accuracy, and coded and analyzed using NVivo (QSR International).

Analysts (MB, KG, and SP) independently coded the first interview and met to discuss the codes and agree on a preliminary codebook (codes and definitions). Then 1 coder (SP) coded the second transcript, adding and revising codes as needed, and discussing any changes in a weekly meeting. The revised codebook was then applied to the next interview, and the codebook was updated until it stabilized, which occurred after 5 transcripts. Then, SP recoded the first transcripts using the final codebook and the 12 remaining transcripts. Three analysts coded the same transcript independently using the final codebook to confirm the reliability of the coding. Select domains from the nonadoption, abandonment, scale-up, spread, and sustainability (NASSS) framework [27] were used to guide organization of the themes identified. NASSS is an evidence-based, theory-informed, pragmatic framework that was developed to help predict and evaluate the success of technology-supported health programs [27]. The framework assumes that it is not individual factors that make or break a technology but a dynamic interaction between them. The framework includes 7 domains, each characterized as simple, complicated, or complex: the condition, the technology, the value proposition, the adopter system, the organization, the wider societal and policy context, and interactions and adaptations over time. Programs characterized as complicated are difficult but not impossible to implement; complex programs are almost always abandoned. The themes that emerged from our data covered many but not all of those defined by NASSS.

### Ethics Approval

The Program for Protection of Human Subjects of the Icahn School of Medicine deemed this study exempt from institutional review board approval (HS# Study-20-02180).

## Results

### Overview

Textbox 1 illustrates the characteristics of the sample studied.

We describe the themes that emerged from interviews presented according to applicable NASSS framework domains (Table 1).

**Sample characteristics.**

**Registered dietitians approached (n=24) and registered dietitians interviewed (n=17)**:

- Response rate: 17/24×100=71%

**Registered dietitians who use Malnutrition Universal Screening Tool (MUST)–Plus across 6 hospitals (n=46) and registered dietitians who were interviewed (n=17):**

- User interviewed rate: 17/46×100=37%

**Number of hospitals represented by registered dietitians interviewed:**

- 5 of 6

**Years worked at the hospital by registered dietitians who use MUST-Plus across 6 hospitals:**

- Mean 4, SD 4; Median 2, range 1-15

**Model versions used by registered dietitians:**

- Versions 1 and 2 (n=9)
- Version 2 only (n=8)

**Table 1:** Nonadoption, abandonment, scale-up, spread, and sustainability domains and study themes.^a^

Domain and questions		Simple, complicated, or complex	Related themes	Key findings and suggested improvements
**Condition**				
	What is the nature of the condition?	Simple—malnutrition and its effects are well characterized in the literature	N/A^b^	N/A
**Organization**				
	How ready is the organization for this technology-supported change? What changes will be needed in team interactions and routines? What work is involved in implementation and who will do it?	Simple—high tension for change, good innovation-system fit, widespread support Complicated—new teams routines or care pathways that align readily with established ones Complicated—some work needed to build shared vision, engage staff, enact new practices, and monitor impact	Workflow Screening and diagnosis Provider communication and trust	Changes to workflow were minimal and well received One screening tool preferred over multiple Pathways for provider communication established and trust high
**Technology**				
	What are the key features of the technology?	Simple—MUST-Plus is embedded in Epic and enhancements have been made to improve algorithm and interface	Enhancements in predictive tool	Improved model accuracy Improved interface to make user friendly and fit workflow
**Value proposition**				
	What is the desirability, efficacy, safety, and cost-effectiveness (demand-side)?	Simple and complicated—technology is desirable, safe, and more effective than previously used screeners, but cost-effectiveness has not yet been fully studied	Usefulness Accuracy and trust	Improved model accuracy which improved usefulness Further improvements possible—wait for missing data, examine algorithmic bias and adjust as necessary
**Adopter system**				
	What changes in staff roles, practices, and identities are implied?	Complicated—existing staff must learn new skills and new staff be appointed	Understanding Patient communication, workload, staffing	Continued education needed as sites are added and staff turnover Communication unchanged—helps prioritize who to see Consider staff workload, especially as algorithm is learning
**Wider context**				
	What is the political, economic, regulatory, professional, and sociocultural context for program rollout?	Complicated—professional stakeholders not yet committed affecting the ability to modify regulatory requirements. If successful, institutions might patent technology for revenue generation The profit, if invested in further improvement, might make the tool available to all beneficiaries	N/A	N/A
**Embedding and adapting over time**				
	How much scope is there for adapting and coevolving the technology and service over time?	Simple—strong scope for adapting and embedding the technology as local need or context changes	Dissemination Suggested opportunities for improvement	Methods for teaching about the tool may need to be adjusted

^a^NASSS is an evidence-based, theory-informed, pragmatic framework that was developed to help predict and evaluate the success of technology-supported health programs [24]. The framework assumes it is not individual factors that make or break a technology, but a dynamic interaction between them. The framework includes 7 domains, each characterized as simple, complicated, or complex: the condition, the technology, the value proposition, the adopter system, the organization, the wider societal and policy context, and interactions and adaptations over time. Programs characterized as complicated are difficult but not impossible to implement; complex programs are almost always abandoned.

^b^N/A: not applicable.

### NASSS Framework Domain: Organization

#### Theme: Workflow

**Figure 1:**
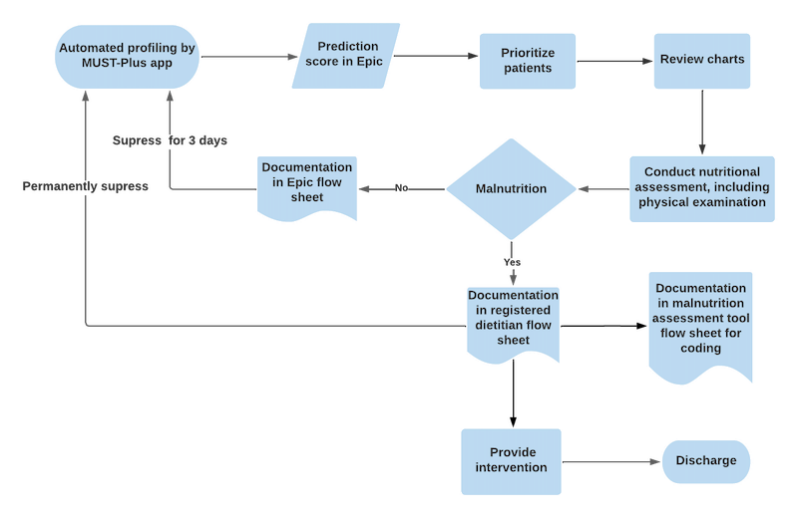
Malnutrition Universal Screening Tool (MUST)–Plus score within the workflow of registered dietitians in this study.

RDs described their flow of work and where the MUST-Plus predictive score fits into this flow (Figure 1). The day typically begins by checking the predictive score in Epic [22] along with the results from other tools (discussed in the screening and diagnosis section below). Then RDs prioritize how they will see patients (usually a combination of risk and location) and review the patient chart for additional information (as necessary). Next RDs meet with the patient to collect pertinent nutritional information such as appetite and weight loss and conduct a physical examination. RDs then document their findings in various flow sheets and provide patient interventions as needed. Most RDs meet with several patients before documenting their findings in Epic. Finally, if the patient remains in the hospital, the predictive score is run again the next day, and follow-up visits may ensue.

#### Theme: Screening and Diagnosis

RDs mentioned 2 other tools being used in tandem with the MUST-Plus predictive tool to screen for malnutrition. One hospital used MUST [20,21] and others used The Malnutrition Screening Tool [28], both consisting of several questions completed by a clinician that determine the risk of malnutrition based on a sum score. Malnutrition was primarily diagnosed using the nationally recognized ASPEN (American Society for Parenteral and Enteral Nutrition) criteria [29-31]. GLIM (Global Leadership Initiative on Malnutrition) criteria [32] were used during the peak of the COVID-19 pandemic to diagnose patients because they relied less heavily on direct patient encounter.

#### Theme: Provider Communication and Trust

The majority of RDs reported that when they found a patient is malnourished, they communicated their findings via Epic [22] Secure Chat with the providers who are responsible for the official malnutrition diagnosis. A few RDs noted that phone or in-person communication occurred sometimes, especially for a patient they were very concerned about. There was a general sense that clinicians trusted and valued RD assessments and that relationships between RDs and clinicians were positive. In a few instances, physicians, particularly older physicians, did not place high value on nutrition and questioned RD assessments, but this was not common at any hospital. In a few other cases, younger physicians asked for more information about RD assessments. RDs saw these inquiries as opportunities for education and were not viewed as physicians lacking trust. One area that required education among physicians was the “old school” belief that someone with low albumin is always malnourished when this is not supported in the literature.

### NASSS Framework Domain: Technology (Theme: Enhancements in the Predictive Tool)

Staff at all hospitals remembered the tool being tested and improving in accuracy over time, particularly between the pilot and go-live date, and between the first and second versions. MUST-Plus was improved over time based on RDs’ feedback. For example, color coding was added to the Epic interface to readily identify patients at higher risk. “Yes or No” boxes were also added for when RDs close out a patient encounter with their follow-up comments. Yes or No selections are fed back into the tool to improve it. To improve workflow, “N/A” was added for cases RDs do not anticipate being able to gather the information needed to determine whether the patient met criteria or cases where malnutrition assessment is not appropriate (ie, hospice patients). Finally, “requires reassessment” was added to flag a patient for quick reassessment if the RD was unable to gather the information needed to determine whether the patient met the criteria for malnutrition diagnosis.

### NASSS Framework Domain: Value Proposition

#### Theme: Usefulness

All RDs reported that the predictive score was useful in their work by identifying patients that needed to be seen and prioritizing their work, but the degree of usefulness varied by the respondent. RDs at the original site tended to think it was more useful. Many RDs noted that the score aided in identifying patients that would not otherwise be seen or would have been seen with a delay of a few days. One RD noted “…we’re catching these patients that we wouldn’t necessarily see until…[day] six.” Another RD stated, “I think it has helped us catch a handful of patients who wouldn't normally maybe been seen high risk.”

The score was seen among many as an objective assessment. As 1 RD stated, “The reason the malnutrition predictive model is helpful is because it's pulling objective data basing whether or not they're high risk based on objective criteria that's in the chart.” Despite an overall belief that the score is valuable, it was noted repeatedly that the score was not used in isolation to diagnose malnutrition. It is one of many inputs RDs used including other screening tools, the patient chart, and their own assessments.

#### Theme: Accuracy

Accuracy refers to how often the patients who are flagged by MUST-Plus as at risk for malnutrition end up being malnourished upon RD assessment. RDs were not asked to track accuracy prior to the interview; thus, information reported is RD’s perception of accuracy based on their experience. RD perception is valuable because if perceived accuracy is low, trust in the score may also be low and scores may be ignored. Perceived accuracy varied from 50% to 70%. Among RDs who had experience working with 2 versions of the tool, all noted that accuracy improved over time and they were grateful for fewer false alarms as it impacted workload. As 1 RD noted, “when it first got rolled out, it was not as specific... we were getting a high score on a lot of our patients, but now it's more specific.” Another RD stated, “In general, the model is stronger than when it first started... initially a good portion of the high [predictive] scores end up being malnourished, as opposed to when it started.”

RDs noted that human error in data entry was 1 factor that led to an inaccurate score. They also noted that the score runs at set timepoints during the day, and for some patients, key data may not yet be available, which can result in inaccurate scores. Even when data were present and correct, other factors reportedly caused the predictive score to be wrong. Inaccuracy was noted among patients at weight extremes—either very low (often young adults, elderly, or Asian patients) or very high, as well as among amputees (because BMI calculations are not meaningful in this population). Albumin levels, fluid overload, anemia from a bleed, and low platelets due to a transplant were other factors reported by RDs across hospitals to be associated with false positives. Some RDs spoke of the need for more autonomy to exercise their professional judgment and override situations where the tool may be inaccurate instead of seeing all patients identified as high risk by MUST-Plus. Many RDs stated that they use the hover tool to check the factors contributing to the score when they question it, and often this double-checking enhanced understanding of an unexpected extreme score.

### NASSS Framework Domain: Adopter System

#### Theme: Understanding

All RDs could articulate a basic understanding of the factors that may contribute to the predictive score, but some, particularly those at MSH and clinical nutrition coordinators, seemed to have a deeper understanding of how the tool worked. All RDs had knowledge of the interface’s hover tool (although not all used it) and understood that the factors listed in it were those that contributed the most to the score. Some thought the factors were always the same in different orders, but others knew they changed based on the patient. Whether RDs understood that the factors listed may just be associated with the score rather than causally linked is unknown; however, many raised questions suggesting that knowledge of how the tool works may enhance understanding of the score. As 1 noted: “I wish I knew exactly how it worked or how the algorithm captures these patients.” Textbox 2 illustrates questions RDs had about MUST-Plus, illustrating the depth of understanding.

**Types of questions registered dietitians asked about the predictive tool.**

- What exactly determines a high score?
- What exactly changed between versions 1 and 2 of the models other than raising the threshold?
- Do factors like albumin, platelets, and hemoglobin all carry the same weight in the model?
- Could the threshold for platelets and hemoglobin be raised? (patients may be at risk for bleeding but may not be malnourished)
- [after rollout] How does diagnosing the patients with yes or no malnutrition impact the model? Does the model learn from the diagnosis? (ie, score was high but the diagnosis was *not* malnutrition, does the model improve?). When does it get incorporated (how quickly)?
- Can weight or weight loss from a previous admission be found or input from Epic to aid in calculating percentage weight loss?

#### Theme: Patient Communication, Workload, and Staffing

RDs reported that communication with patients did not change with the implementation of the predictive tool and they do not mention the score when they meet with patients. Most also noted that the amount of time they spend with patients was about the same since implementation. As 1 RD noted, “[The score] is less defining how long we're spending with the patients and more defining who we are seeing.”

Most RDs mentioned that the number of patients they saw had increased since the predictive tool was implemented and that adequate staffing was important. A few RDs discussed ongoing staffing shortages amid the pandemic and noted the score added to the workload on an already busy day. Some RDs described cases where workload increased because MUST-Plus misidentified patients as high-risk, even though upon chart review, RDs could tell these patients were likely low-risk. As 1 RD noted:

sometimes we’re reading through the chart, we’re looking at a patient’s BMI and we don’t think they’re going to be malnourished. So sometimes those patients do have the high score, but their BMI is very high and we’re reading through the chart and we’re where we have a low suspicion of malnutrition. So sometimes, it can add to the workload in a way that patient is not, probably not very high risk and they could have waited.

Even though these cases led to additional work, RDs repeatedly noted that they accepted these shortcomings and valued the score overall.

### NASSS Framework Domain: Embedding and Adapting Over Time

#### Theme: Dissemination

As noted previously, the predictive score was tested first at MSH. At this site, a working group composed of data scientists, RDs, and hospital administrators was formed to guide implementation. Outside of MSH, most RDs noted that they learned about the predictive score from supervisors or managers, showing the score in Epic and teaching its interpretation and the timing with which follow-up is needed. There was a difference in depth of tool understanding between the supervisors or managers who were trained by the data science team and those who supervisors or managers trained.

#### Theme: Suggested Opportunities for Improvement

RDs discussed several ways the predictive tool could be improved. Suggestions involved improving accuracy by changing the factors such as low albumin that feed the algorithm or modifying the weight certain factors play. Many RDs wanted more education about how the tool works. Running the score only when all laboratory data were present was also suggested as an improvement. Many RDs noted that a single screening tool would be preferred to the use of multiple tools: “Right now it feels like we have so many screening tools, if there was [the] ability to kind of streamline it to using just one [it would be great].”

## Discussion

### Principal Results

This qualitative postimplementation evaluation identified many themes that may be useful to the expansion of MUST-Plus in other settings and to the implementation of other ML tools. Using the NASSS framework to organize our themes helped reveal that for most domains examined, MUST-Plus was either simple or complicated, but never complex and this may be responsible for its implementation success. The MUST-Plus predictive tool was well received among the RDs who rely on it in their everyday work. Reported effects on workflow were positive; the tool provided a way to prioritize patients and this helped shape work for the day. Positive reception of the tool among staff is likely due to the involvement of RDs in the tool design, enhancements, and implementation. Although workflow remained largely uninterrupted, the reported workload did increase, particularly when the score was less accurate with version 1 of the tool. Alert fatigue and the importance of considering the clinical burden of CDSS are well established in the literature [33-38]. Our study highlights the value of testing and improving tools over time to reduce false positives and minimize staff burden and the importance of ensuring adequate staffing while tools are being implemented.

Importantly, RDs outside of the original hospital and nonsupervisory staff had more questions about how the tool worked and were curious to learn more. The importance of explainability of artificial intelligence (AI) output among stakeholders is highlighted in the recently published AI principles set forth by the American Medical Informatics Association [39]. Although RDs continued to be engaged as the MUST-Plus was rolled out at different hospitals and excitement for the tool was high, the depth of tool understanding did not always transfer to those trained by supervisors or managers. Our study findings suggest that ongoing involvement and training with a broader set of stakeholders at each new site is critical in addition to early involvement, especially as staff turnover. Furthermore, modifying the timing and composition of stakeholders involved in implementation is also important. This would require close coordination between RD staff and the data science team, which could be challenging.

RDs hoped a single streamlined screening tool could be used in the future instead of relying on many tools. Staff burden is an important consideration when implementing CDSS and the ultimate goal should be to replace less efficient tools with more efficient ones. Decisions about removing overlapping CDSS or tools can be brought to a team discussion to weigh risks and benefits.

RDs discussed potential bias in MUST-Plus scores, noting that scores were less accurate for certain populations such as Asian people and amputees. Fairness and the absence of bias are among the principles for the use of AI in health care outlined by the American Medical Informatics Association [39] as algorithmic bias is well established in the literature [40-44]. Such bias should be considered when developing ML models that feed CDSS so existing inequity is not exacerbated. This highlights the value of qualitative research because RDs perceived bias may result in not trusting the model’s output, whether or not true bias exists. Qualitative inquiry during implementation is therefore important to assess the perceptions of users. We urge others implementing ML-based CDSS to consider bias, gather ongoing, qualitative feedback from the staff who rely on ML tool output, and tweak algorithms as needed.

### Limitations

Our study has some limitations. Interviews were conducted after CDSS go-live dates; thus, some of the planning and preparation processes were not captured or may have been captured in less detail. The study took place in a single health system, which may affect the applicability of our findings to other settings. We did not interview providers about the downstream effects of the CDSS on their interactions with RDs or malnutrition diagnosis in their patients. Data scientists who developed the tool, while involved in the study as experts, were excluded from interviews. This decision was driven by the fact that data scientists’ familiarity with CDSS implementation in clinical practice was limited. We also lacked demographic information on the RDs we interviewed other than the number of years they worked at Mount Sinai and factors such as age could affect perceived tool usefulness. We did, however, select RDs with a wide range of years of experience at Mount Sinai, which may be a proxy for age. Finally, the use of the NASSS framework helped us organize the themes that emerged from our data and we found value in this, but our interview guide was not developed using the framework, and thus not all domains were covered by our data. Future studies could focus on questions by domain so data are collected for all areas.

### Conclusions

Many important lessons were learned in this evaluation of the MUST-Plus ML–based CDSS. Our findings highlight that ML tools will not replace clinicians but augment their capabilities, and therefore active involvement of clinicians in all stages of ML tool development and implementation is critical. The use of qualitative methods was integral to identifying the need for involving a broader set of stakeholders as sites were added, continuous training as staff turnover, and the potential for algorithmic bias. Possible solutions need to be developed in collaboration with clinicians and patients who are affected by these decisions (eg, provide nutritional intervention). The success of this work provides motivation to others for the need of evaluation to increase the value of CDSS.

## Appendix

Multimedia Appendix 1 Semistructured interview guide.

## Abbreviations

AI: artificial intelligence
CDSS: clinical decision support systems
ML: machine learning
MSH: Mount Sinai Hospital
MUST: Malnutrition Universal Screening Tool
NASSS: nonadoption, abandonment, scale-up, spread, and sustainability
RD: registered dietitian

## References

[1] Taylor RA, Haimovich AD (2021). Machine learning in emergency medicine: keys to future success. Acad Emerg Med.

[2] Cabitza F, Rasoini R, Gensini GF (2017). Unintended consequences of machine learning in medicine. JAMA.

[3] Vasey B, Ursprung S, Beddoe B, Taylor EH, Marlow N, Bilbro N, Watkinson P, McCulloch P (2021). Association of clinician diagnostic performance with machine learning-based decision support systems: a systematic review. JAMA Netw Open.

[4] Schwartz JM, Moy AJ, Rossetti SC, Elhadad N, Cato KD (2021). Clinician involvement in research on machine learning-based predictive clinical decision support for the hospital setting: a scoping review. J Am Med Inform Assoc.

[5] Kia A, Timsina P, Joshi HN, Klang E, Gupta RR, Freeman RM, Reich DL, Tomlinson MS, Dudley JT, Kohli-Seth R, Mazumdar M, Levin MA (2020). MEWS++: enhancing the prediction of clinical deterioration in admitted patients through a machine learning model. J Clin Med.

[6] Parchure P, Joshi H, Dharmarajan K, Freeman R, Reich DL, Mazumdar M, Timsina P, Kia A (2020). Development and validation of a machine learning-based prediction model for near-term in-hospital mortality among patients with COVID-19. BMJ Support Palliat Care.

[7] Cheng FY, Joshi H, Tandon P, Freeman R, Reich DL, Mazumdar M, Kohli-Seth R, Levin M, Timsina P, Kia A (2020). Using machine learning to predict ICU transfer in hospitalized COVID-19 patients. J Clin Med.

[8] Moskowitz G, Egorova NN, Hazan A, Freeman R, Reich DL, Leipzig RM (2020). Using electronic health records to enhance predictions of fall risk in inpatient settings. Jt Comm J Qual Patient Saf.

[9] Timsina P, Joshi HN, Cheng FY, Kersch I, Wilson S, Colgan C, Freeman R, Reich DL, Mechanick J, Mazumdar M, Levin MA, Kia A (2021). MUST-Plus: a machine learning classifier that improves malnutrition screening in acute care facilities. J Am Coll Nutr.

[10] Jameson JL, Fauci AS, Kasper DL, Hauser SL, Longo DL, Loscalzo J (2018). Malnutrition and nutritional assessment. Harrison's Principles of Internal Medicine, 20th edition.

[11] de Luis DA, Izaola O, Cuellar L, Terroba MC, Cabezas G, Rojo S, Aller R, Sagrado MG (2006). Nutritional assessment: predictive variables at hospital admission related with length of stay. Ann Nutr Metab.

[12] Culebras JM (2013). Malnutrition in the twenty-first century: an epidemic affecting surgical outcome. Surg Infect (Larchmt).

[13] Kruizenga H, van Keeken S, Weijs P, Bastiaanse L, Beijer S, Huisman-de Waal G, Jager-Wittenaar H, Jonkers-Schuitema C, Klos M, Remijnse-Meester W, Witteman B, Thijs A (2016). Undernutrition screening survey in 564,063 patients: patients with a positive undernutrition screening score stay in hospital 1.4 d longer. Am J Clin Nutr.

[14] Correia MITD, Waitzberg DL (2003). The impact of malnutrition on morbidity, mortality, length of hospital stay and costs evaluated through a multivariate model analysis. Clin Nutr.

[15] Kubrak C, Jensen L (2007). Malnutrition in acute care patients: a narrative review. Int J Nurs Stud.

[16] Curtis LJ, Bernier P, Jeejeebhoy K, Allard J, Duerksen D, Gramlich L, Laporte M, Keller HH (2017). Costs of hospital malnutrition. Clin Nutr.

[17] Browning M, Phillips W (2017). A clinician's guide to defining, identifying and documenting malnutrition in hospitalized patients. Pract Gastroenterol.

[18] (2020). Malnutrition in hospitalized adults: research protocol October 30, 2020. Effective Health Care Program.

[19] National Association of Community Health Centers.

[20] Chao PC, Chuang HJ, Tsao LY, Chen PY, Hsu CF, Lin HC, Chang CY, Lin CF (2015). The malnutrition universal screening tool (MUST) and a nutrition education program for high risk cancer patients: strategies to improve dietary intake in cancer patients. BAPEN.

[21] Stratton RJ, Hackston A, Longmore D, Dixon R, Price S, Stroud M, King C, Elia M (2004). Malnutrition in hospital outpatients and inpatients: prevalence, concurrent validity and ease of use of the 'malnutrition universal screening tool' ('MUST') for adults. Br J Nutr.

[22] Epic.

[23] Oracle Cerner.

[24] SCC Soft Computer.

[25] GE Healthcare.

[26] Creswell JW (2015). A Concise Introduction to Mixed Methods Research.

[27] Greenhalgh T, Wherton J, Papoutsi C, Lynch J, Hughes G, A'Court C, Hinder S, Fahy N, Procter R, Shaw S (2017). Beyond adoption: a new framework for theorizing and evaluating nonadoption, abandonment, and challenges to the scale-up, spread, and sustainability of health and care technologies. J Med Internet Res.

[28] Malnutrition Screening Tool (MST).

[29] White JV, Guenter P, Jensen G, Malone A, Schofield M, Academy Malnutrition Work Group, A.S.P.E.N. Malnutrition Task Force, A.S.P.E.N. Board of Directors (2012). Consensus statement: Academy of Nutrition and Dietetics and American Society for Parenteral and Enteral Nutrition: characteristics recommended for the identification and documentation of adult malnutrition (undernutrition). JPEN J Parenter Enteral Nutr.

[30] White JV, Guenter P, Jensen G, Malone A, Schofield M, Academy Malnutrition Work Group, A.S.P.E.N. Malnutrition Task Force, A.S.P.E.N. Board of Directors (2012). Consensus statement: academy of nutrition and dietetics and american society for parenteral and enteral nutrition: characteristics recommended for the identification and documentation of adult malnutrition (undernutrition). JPEN J Parenter Enteral Nutr.

[31] Malone A, Hamilton C (2013). The academy of nutrition and dietetics/the american society for parenteral and enteral nutrition consensus malnutrition characteristics: application in practice. Nutr Clin Pract.

[32] Cederholm T, Jensen GL, Correia MITD, Gonzalez MC, Fukushima R, Higashiguchi T, Baptista G, Barazzoni R, Blaauw R, Coats A, Crivelli A, Evans DC, Gramlich L, Fuchs-Tarlovsky V, Keller H, Llido L, Malone A, Mogensen KM, Morley JE, Muscaritoli M, Nyulasi I, Pirlich M, Pisprasert V, de van der Schueren MAE, Siltharm S, Singer P, Tappenden K, Velasco N, Waitzberg D, Yamwong P, Yu J, Van Gossum A, Compher C, GLIM Core Leadership Committee, GLIM Working Group (2019). GLIM criteria for the diagnosis of malnutrition - a consensus report from the global clinical nutrition community. Clin Nutr.

[33] Jung SY, Hwang H, Lee K, Lee HY, Kim E, Kim M, Cho IY (2020). Barriers and facilitators to implementation of medication decision support systems in electronic medical records: mixed methods approach based on structural equation modeling and qualitative analysis. JMIR Med Inform.

[34] Espel-Huynh HM, Goldstein CM, Finnegan OL, Elwy AR, Wing RR, Thomas JG (2021). Primary care clinicians' perspectives on clinical decision support to enhance outcomes of online obesity treatment in primary care: a qualitative formative evaluation. J Technol Behav Sci.

[35] Isaac T, Weissman JS, Davis RB, Massagli M, Cyrulik A, Sands DZ, Weingart SN (2009). Overrides of medication alerts in ambulatory care. Arch Intern Med.

[36] Nanji KC, Slight SP, Seger DL, Cho I, Fiskio JM, Redden LM, Volk LA, Bates DW (2014). Overrides of medication-related clinical decision support alerts in outpatients. J Am Med Inform Assoc.

[37] Topaz M, Seger DL, Slight SP, Goss F, Lai K, Wickner PG, Blumenthal Kimberly, Dhopeshwarkar Neil, Chang Frank, Bates David W, Zhou Li (2016). Rising drug allergy alert overrides in electronic health records: an observational retrospective study of a decade of experience. J Am Med Inform Assoc.

[38] Getty DJ, Swets JA, Pickett RM, Gonthier D (1995). System operator response to warnings of danger: a laboratory investigation of the effects of the predictive value of a warning on human response time. J Exp Psychol Appl.

[39] Solomonides AE, Koski E, Atabaki SM, Weinberg S, McGreevey JD, Kannry JL, Petersen C, Lehmann CU (2022). Defining AMIA's artificial intelligence principles. J Am Med Inform Assoc.

[40] Obermeyer Z, Powers B, Vogeli C, Mullainathan S (2019). Dissecting racial bias in an algorithm used to manage the health of populations. Science.

[41] Sun W, Nasraoui O, Shafto P (2020). Evolution and impact of bias in human and machine learning algorithm interaction. PLoS One.

[42] O'Reilly-Shah VN, Gentry KR, Walters AM, Zivot J, Anderson CT, Tighe PJ (2020). Bias and ethical considerations in machine learning and the automation of perioperative risk assessment. Br J Anaesth.

[43] Barocas S, Selbst AD (2016). Big data's disparate impact. Berkeley Law.

[44] Skeem JL, Lowenkamp CT (2016). Risk, race, and recidivism: predictive bias and disparate impact. Criminology.

