# Supplementary material for: Implementing a Machine Learning Screening Tool for Malnutrition: Insights From Qualitative Research Applicable to Other Machine Learning–Based Clinical Decision Support Systems
Source: JMIR Form Res. 2023 Jul 13;7:e42262. doi: 10.2196/42262 (PMC10375393; doi:10.2196/42262)
Supplement: Multimedia Appendix 1 [file formative_v7i1e42262_app1.docx]

Appendix

MUST-Plus Evaluation: Understanding Implementation

Semi-Structured Interview Guide

Thank you for taking time to talk with me today. We know how very busy you are, and will try to not take too much of your time. As you know, **we are trying to better understand how the Nutrition Predictive Model/Score has been implemented at Mount Sinai**. Often when models are implemented, no one records ***how*** they were implemented and we lose information about what factors helped make them successful or what could have been done better to make implementation smoother. Registered dietitians like you play a key role in implementing the Nutrition Predictive Model. Understanding how you work with the output from the model can help us make sense of the data we look at, it can help improve the model at Mount Sinai, and may help other institutions who want to implement a similar model. So, your input, positive or negative, is very important.

We would like to record this interview if you don’t mind so we can capture your words exactly. When I start the recording, I will try to avoid using your name so your information remains confidential. If I do use your name or if you mention any identifying information, we will remove it from the transcript so the data cannot be traced directly to you. Feel free to skip any questions or ask me to pause the recording if you would like to say something off the record. Do you have any questions before we begin? Is it okay for me to start the recording?

**<***Start recording***>**

(Do not repeat questions if the interviewee already answered them. Feel free to move across topics if needed.)

**Background**

- How many years have you been a registered dietitian/ nutritionist?
- [When did you first start working as an RD at Mount Sinai?]
- What type of patients do you typically work with? (cancer, surgical, etc)

**Workflow**

- **Interactions with patients:** Please walk me through a typical day at work. (Where does the Malnutrition Predictive Model/Score fit within your day/week?)

- What do you typically tell patients about why you are seeing them?
- Do you mention the Predictive Model/Score?

- **What information do you gather from the patients** you see on your list? Physical exam?
  - How much time, on average do you spend assessing each patient?
  - If RD worked at Mount Sinai before MUST+ ask: how about before Sept 2018? (before implementation)
  - How has your routine changed since **Covid**?
  - Some RDs have mentioned the use of the **GLIM criteria**? Do you currently use them? Did you use them before Covid?

- **Data**: When and where and how do you enter data? (EPIC)
  - What do you do with the information you gather from patients? Where are data entered? When are data entered? After each patient encounter? In batches?

- Tell me about **how you communicate your patient assessments with the providers** who are responsible for documenting malnutrition.

- Are there **particular types of patients** who you’re more likely to discuss with the treatment team? (e.g., severe malnutrition, comorbidities?)
- **How often in a day do you communicate**? After each patient? In batches? During interdisciplinary rounds?
- **How receptive are providers** to your assessments? How often do they disagree with your assessments? How could communication be improved?

**Training/Rollout and Model Accuracy**

- How did you learn what to do once you received the Predictive Model/Score? Who taught you? How?

- Has anything changed about the Predictive Model/Score since you were trained? What? When did this change? What prompted the change? What do you think of the change?

**Predictive Model/Score Feedback**

- **Knowledge/ usability**: Do you understand what the Predictive Model/Score is based on? If no, would you have liked to know? Have you seen the publication from Mount Sinai team on the Predictive Model/Score (they call it MUST+).
  - Can you tell me about the reasons why some patients are predicted positive (Peach color)?
  - When you hover over a predictive score – what does the additional information in the hover bubble tell you?  Is it helpful to you?
  - Would you prefer more information about *HOW* the model made a decision? Would you rather have a model that gives little information but is correct more often?

- Tell me about the **usefulness** of Predictive Model/Score in your everyday work?

- How does it help you in your work?
- How does it hinder you?
- How has it affected **your communication** or interactions with patients?

- **Perception of model accuracy**: [For staff who have experienced different versions of the model] Do you think the model has improved accuracy since it was first introduced?

- **Trust**: How much do you trust the Predictive Model/Scores you receive?
  - Can you tell me about a time when you received a predictive score that was completely inaccurate? What do you think caused this? What did you do?

- Can you talk about a time where the Predictive Model gave you information that you wouldn’t have known otherwise?

- Do you think the Predictive Model produces correct score for different kinds of patients, e.g. **race, gender, age**? Do you think the model is **biased**?

Wrap-up/ Closing:

- **Improvements**: If you could change one thing about the Predictive Model/Score at Mount Sinai what would it be?

- **Other** **potential respondents**: If I could speak to one other person at your hospital that has been critical to the implementation of the Predictive Model/Score who would that be? [name and role]

- **Advice**: If another hospital was considering implementing the same Predictive Model/Score what advice would you give them?
